# BROADBAND ACCESS, SOCIAL CONNECTEDNESS, AND SELF-REPORTED HEALTH IN A RURAL COMMUNITY SAMPLE OF OLDER ADULTS

**DOI:** 10.1093/geroni/igad104.2185

**Published:** 2023-12-21

**Authors:** Tim Killian, Betsy Garrison

**Affiliations:** University of Arkansas, Fayetteville, Arkansas, United States; University of Arkansas, Fayetteville, Arkansas, United States

## Abstract

Social connections provide older persons with access to important resources supporting their physical and psychological health. People aging in rural areas may have more challenges to maintaining and developing social connectedness because of geographic separation with friends, neighbors, and family members. One way to mitigate the risk of reduced social connectedness is by using internet-enabled technologies to facilitate social connections. However, these solutions are also limited by internet access, stability, and questions about whether or not technology enabled connections are able to meet needs for social connections. The objective of this study was to examine the role of internet access to (a) social connectedness and (b) self-rated health (SRH) and direct and indirect pathways from internet access to SRH. We used data collected in 2020 during the height of COVID-19 pandemic lockdowns from a random sample of about 450 people aged 50 years old or older living in rural counties in Arkansas. Path analyses were used to regress participants’ self-reported health on social connectedness and examine direct and indirect pathways from internet access to health. Interestingly, we found that internet access was positively related to self-reported health, but was fully mediated through social connectedness. Moreover, these relationships remained significant even after controlling for variabilities in income. Although more research is needed, these results suggest that maintaining social connections using the internet was an effective approach to support the health of the older rural persons and has policy implications for maintaining and continuing to increase stable broad-band technologies for rural areas.

